# Value of Combined Diagnosis for Choroidal Lymphoma: A Case Report

**DOI:** 10.3390/curroncol29110695

**Published:** 2022-11-17

**Authors:** Ming Yang, Taoran Zhang, Bojing Yan, Yingxiang Huang

**Affiliations:** Department of Ophthalmology, Beijing Friendship Hospital, Capital Medical University, Beijing 100050, China

**Keywords:** intraocular lymphoma, choroidal lymphoma, tumour, lymphohematopoietic tissue, choroid

## Abstract

Intraocular lymphoma (IOL) comprises a group of malignant tumours originating from lymphohematopoietic tissues that have a poor prognosis. These tumours predominantly occur in the vitreous and retina but are rarely found in the choroid. A few case reports and case series of choroidal lymphoma (CL) have been reported in the literature. CL is prone to misdiagnosis and incorrect treatment because it often mimics other intraocular diseases such as uveitis. This may seriously affect localisation of the primary lesion and delay treatment, which may even affect the patient’s survival. Herein, we report a case of CL and propose the combination of characteristic ophthalmic imaging with systemic imaging and aqueous humour detection to establish a robust basis for the early diagnosis of CL.

## 1. Introduction

Intraocular lymphoma (IOL) is a group of malignant tumours that originate from the lymphohematopoietic tissue [1]. The incidence of IOL has risen in recent years, and the overall estimated incidence rate is 1.86% of ocular malignant tumours [2]. The World Health Organization further classifies IOL into primary vitreous retinal lymphoma, choroidal lymphoma (CL), ciliary body lymphoma, and iris lymphoma [3]. Histopathologically, CL is predominantly B-cell non-Hodgkin’s lymphoma, and can be classified as primary or secondary CL on the basis of systemic evaluation [4,5]. The first case of IOL was reported by Cooper et al. [6], and in 1968, Vogel et al. [7] described four cases of reticulosarcoma involving the retina for the first time and confirmed the existence of a correlation between central nervous system lymphoma and IOL. In 1983, Nelson et al. first reported the overall incidence of ocular involvement in cases of fatal systemic malignant lymphomas (4/60) [8]. In 1985, Leff et al. [9] found an unusual case in which the choroidal tumours manifested early during the course of the patient’s illness, before the first presentation of systemic signs or symptoms.

Lymphomas are rarely located in the choroid, and there is a paucity of case reports and case series on CL in the literature. The diagnosis of CL is difficult because it is a rapidly progressive tumour that mimics other diseases. Furthermore, performing histopathological biopsy of CL is an arduous task. Therefore, we reported this case to explore the specific clinical manifestations of CL to enable early diagnosis.

## 2. Case Report

A 56-year-old man was admitted to the Department of Ophthalmology of the Capital Medical College, Beijing Friendship Hospital (Beijing, China) in August 2019 with a history of bilateral progressive vision loss for 1 week. Simultaneously, the patient experienced defects in the visual field, and the symptoms in the right eye were more severe.

**Figure 1:**
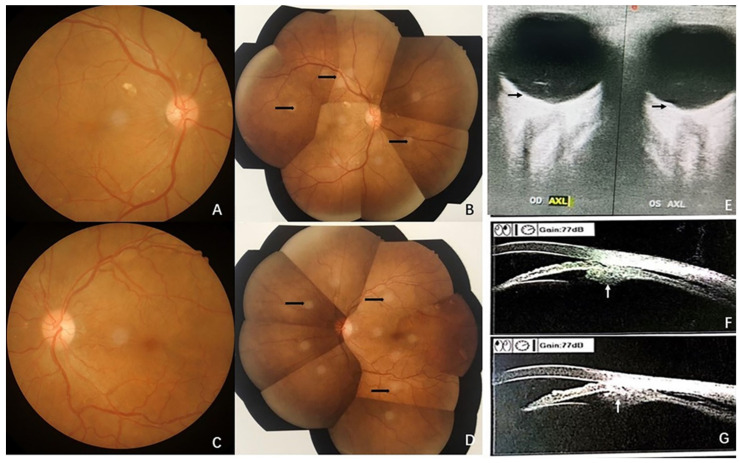
Fundus photos (**A**,**C**) Fundus colour photograph, OD and OS. (**B**,**D**) showed multiple serous retinal detachment (Black arrow). B-scan ultrasonography of both eyes revealed retinal eminence and choroidal oedema (black arrow) in both eyes (**E**) and ultrasound biomicroscopy revealed partial diffuse thickening (white arrow) of the ciliary body and local suprachoroidal effusion (**F**,**G**).

**Figure 2:**
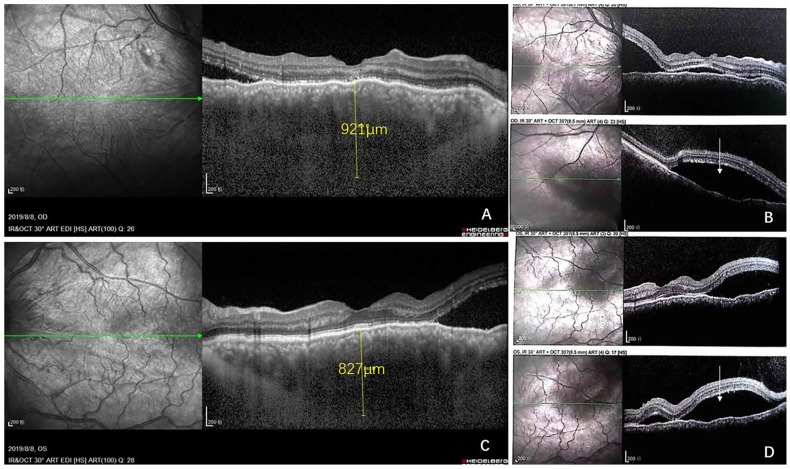
Optical coherence tomography (OCT) showed neuroepithelial detachment. Choroidal thickness was found to be 921 and 882 μm in the right and left eye (**A**,**C**). Significant subfoveal fluid (arrow) in both right and left eye (**B**,**D**).

**Figure 3:**
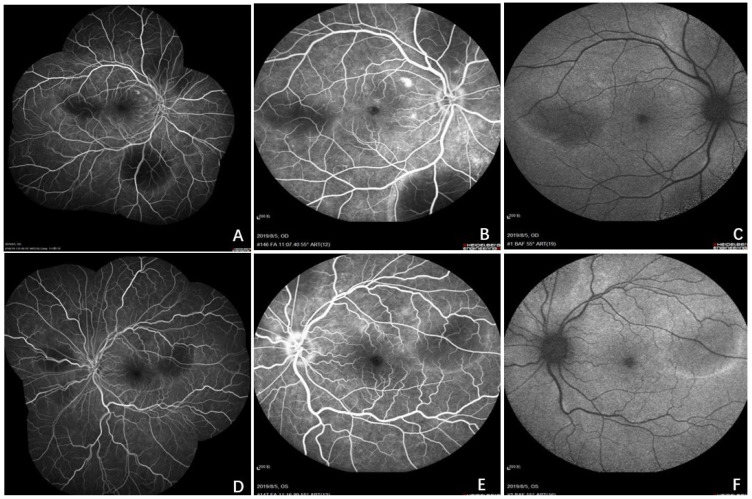
Fluorescein angiography (FA) showed uniform and less than one cloud fluorescence in the later stage. The temporal 1PD of the macular fovea showed effusion under the neuroepithelium, presenting as a liquid dark area (**A**,**D**). Small flakes of spontaneous fluorescence enhancement in the temporal part above the optic disc (**B**,**E**); autofluorescence showed irregular subretinal fluorescein accumulation was observed in the posterior pole in the last phase (**C**,**F**).

**Figure 4:**
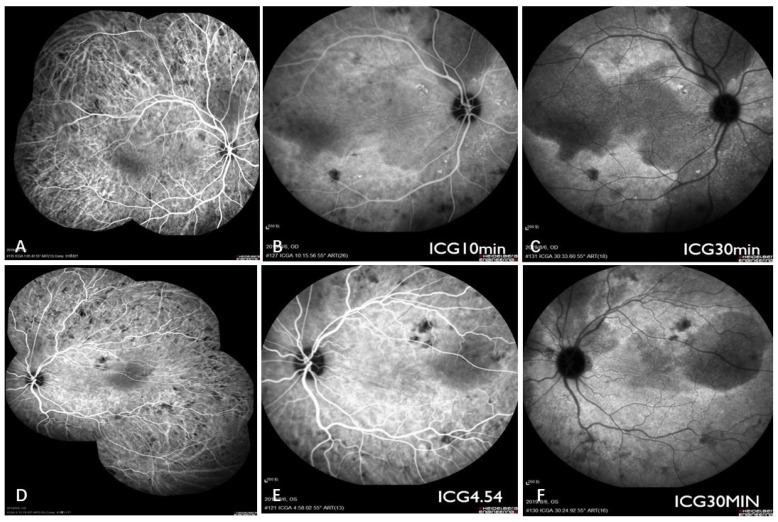
Indocyanine green angiography (ICGA) revealed hypofluorescent spots of different sizes from the early to late phase, corresponding with multiple choroidal nevus-like lesions (**A**,**D**). ICGA in early phase (**B**,**E**). ICGA in late phase (**C**,**F**).

Initial examination revealed a visual acuity of 20/40 and 20/50 in the right and left eyes, respectively. Intraocular pressure and anterior segment examination of both eyes yielded unremarkable results. Fundus examination revealed multiple serous retinal detachments (SRDs) but no vitreous opacity in both eyes. Local flake depigmentation was observed on the nasal side of the optic disc in both eyes and retinal folds were observed at the posterior pole (Figure 1). B-scan ultrasonography of both eyes revealed neuroepithelial detachment, retinal eminence, and thickening of the choroidal layer in both eyes without scleral thickening or sub-Tenon fluid. Ultrasound biomicroscopy (UBM), which combines ophthalmic UBM with high-frequency ultrasonic imaging technology, revealed local suprachoroidal effusion and partial diffuse thickening of the ciliary body (Figure 1). Optical coherence tomography (OCT), which mainly utilises the interference principle of light to yield the tomography of eye tissues, depicted a clear neuroepithelial layer in the retina; no local abnormal structural disorder was observed. The neuroepithelial and pigment epithelial layers showed a wavy contour, with liquid dark areas under the nerve and choroidal thickening in both eyes (Figure 2). Choroidal thickness, as measured by enhanced-depth imaging (EDI) OCT, was 921 and 882 μm for the right and left eyes, respectively (Figure 2). Fluorescein angiography (FA), which involves injection of a fluorescent contrast agent that enters the fundus through a blood vein and use of a fundus camera to photograph the dynamic circulation of the contrast agent in the fundus blood vessels, depicted small flakes of spontaneous fluorescence enhancement in the temporal part above the optic disc in the early stage. A copious amount of high punctate fluorescence appeared below the nose of the optic disc (1.5 PD), but no obvious fluorescence leakage was observed, and local staining was observed in the late stage. The temporal 1 PD of the macular fovea showed effusion under the neuroepithelium, presenting as a liquid dark area (Figure 3). Indocyanine green dye with macromolecular properties is injected into the veins, enabling its circulation within the choroidal vessels, in indocyanine green angiography (ICGA) (similar to FA). ICGA revealed hypofluorescent spots of different sizes from the early to late phase, corresponding to multiple choroidal nevus-like lesions. Thus, the amalgamation of the results of FA and ICGA revealed that the high fluorescence part was located on the temporal side of the disc, corresponding to the local high leakage point of the choroid (Figure 3 and Figure 4).

**Figure 5:**
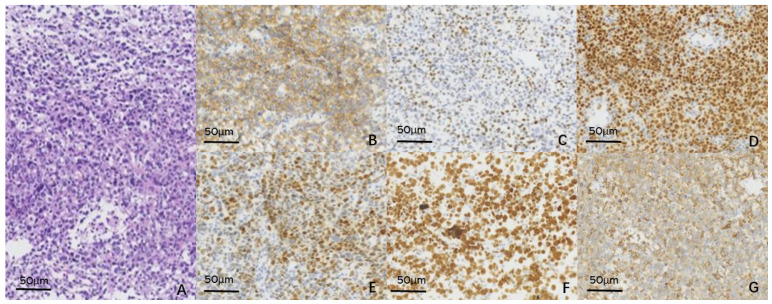
The pathology report of the axillary nodes biopsy confirmed the diagnosis of non-Hodgkin diffuse large B-cell lymphoma. (**A**) The HE staining figure of biopsy. (**B**–**G**) The figure of immunohistochemical staining of CD20, c-myc, PAX-5, MUM1, Ki-67, CD5.

We performed an anterior chamber puncture and analysed the aqueous humour to confirm the diagnosis. The levels of cytokines, namely, interleukin (IL)-10 and IL-6, were measured using enzyme-linked immunosorbent assay or chemiluminescent enzyme immunoassay. The IL-10/IL-6 ratio was 0 (Table 1). We proposed a chorioretinal biopsy; however, the patient refused to undergo this procedure. Systemic evaluation was immediately performed to obtain a definitive diagnosis. Computed tomography (CT) and contrast-enhanced CT revealed multiple enhanced nodules and masses in the liver, spleen, intraperitoneal and retroperitoneal spaces, and double adrenal masses, with multiple lymphadenopathies in the right diaphragmatic angle. The results of complete blood count were as follows: white blood cell count, 5580/mL; haemoglobin, 76 g/L; platelets, 4600/mL; and C-reactive protein, 132 mg/L. Moreover, a peripheral blood smear revealed that the proportion of mature lymphocytes and monocytes was 11% and 20%, respectively. The results of biochemical analysis were as follows: creatinine 171.6 µmol/L; glutamic oxaloacetic transaminase, 430.6 U/L; glutamic oxaloacetic transaminase, 131.9 U/L; lactate dehydrogenase, 5306 U/L; creatine kinase, 48 U/L; and myoglobin, 257 ng/mL. The pathological findings of the left axillary lymph node biopsy included structural destruction of the lymph node, diffuse heterogeneous lymphocyte infiltration, and a source of activated B cells located outside the germinal centre. The histochemistry results were as follows: CD20 (+), Ki67 (+, about 90%), Bcl-6 (+), MUM1 (+), CD5 (+), c-Myc (+), PAX-5 (+), CD21 (−), CD3 (−), CD10 (−), Bcl-2 (−), cyclin D1 (−), CD30 (−), and ALK (−) (Figure 5). The results of serological testing for the human immunodeficiency virus were negative. The combination of imaging features, haematological examination, and histopathological examination of the axillary lymph node puncture and bone marrow specimen resulted in a definitive diagnosis of diffuse large B-cell non-Hodgkin lymphoma (IVB, IPI4). Finally, the diagnosis of CL was clarified.

The patient was transferred to the haematology department for further treatment and administered rituximab 600 mg/D1. Unfortunately, the patient developed acute tumour lysis syndrome, myocardial injury, cardiac insufficiency, renal insufficiency, hyperuricemia, hyperphosphatemia, and respiratory failure, accompanied by recurrent high fever, pulmonary infection, and intestinal fungal infection. In addition, the patient developed anaemia, progressive decline in the platelet count, and high ferritin levels; thus, we were alert to the possibility of hemophagocytic syndrome. Unfortunately, the patient’s family refused to give us permission to perform further invasive investigations and requested that the patient be discharged and treatment ceased.

## 3. Discussion

### 3.1. Brief Review of CL

CL is usually composed of highly or poorly differentiated malignant B-cell proliferation [10]. CL is a subset of uveal lymphoma, which can be classified as primary and secondary lymphoma based on the presence of systemic lymphoma at the time of ocular presentation. A primary choroidal lymphoma (PCL) is defined as a CL in the absence of prior systemic lymphomas or concurrent extraocular lymphomas. PCL is an indolent, low-grade tumour, previously known as reactive lymphoid hyperplasia or uveal pseudotumor, while secondary CL is often a comparatively high-grade lymphoma, characterised by a more rapidly progressive course [11]. In our case, important organs, such as the liver, spleen, and adrenal gland, were involved and the neck and axillary lymph nodes were enlarged. This patient did not exhibit any ocular symptoms of vitreoretinal lymphoma; however, systemic lymphoma was present. The disease progressed rapidly. Lymphoma cells spread to the choroid via blood, causing secondary CL.

The diagnosis of IOL is often challenging, owing to its propensity to masquerade as other ocular diseases. Although the typical manifestations of fundus involvement are uniform, which are accompanied by solid thickening of the choroid, isolated or multifocal choroidal infiltration, SRD, and monocular or binocular onset, CL is often a diagnosis of exclusion that can easily be missed [12,13]. The principal differential diagnoses include amelanotic choroidal melanoma, metastases, and several inflammatory entities such as granulomatous choroiditis, Vogt–Koyanagi–Harada (VKH) disease, birdshot chorioretinopathy, and paraneoplastic retinopathy (PR) [14,15]. Hence, CL can be easily confused with other diseases, consequently leading to misdiagnosis. Therefore, we described the characteristics of CL on B-ultrasonography, UBM, OCT, FA, and ICGA, in order to consider lymphoma as early as possible during the diagnostic process. Presently, the diagnosis of IOL depends on intraocular biopsy, followed by a combination of cytologic evaluation, immunohistochemistry, and flow cytometry [16,17]. Moreover, molecular testing and proteomic analysis can identify novel diagnostic biomarkers for IOL, which have the potential to improve timely diagnoses of IOL and patient outcomes [18]. Hernández-Pons et al. [19] used the IgH Rearrangements Molecular Analysis Kit and monoclonal amplification with three primer mixes (FR1-JH, FR2-JH, and FR3-JH) and diagnosed approximately 90% of all mature B-cell lymphoproliferative processes. They found that polymerase chain reaction-based clonality testing can serve as a valuable tool to confirm the choroidal lymphoproliferative process.

To date, no standardised treatment has been established for CL. The available treatment modalities include observation, radiotherapy, systemic chemotherapy, and systemic immunotherapy (rituximab). Systemic chemotherapy and immunotherapy are typically reserved for systemic disease, while isolated choroidal involvement is treated using radiotherapy [20,21]. The prognosis for survival in patients with PCL is usually good. The prognosis of secondary CL depends on the grade of systemic lymphoma [22]. The overall survival at 2 years after external-beam radiotherapy for primary intraocular lymphoma was 94%, which was closely followed by orbital radiotherapy with a median dose of 36 Gy in the event of relapse [23]. In addition, ultra-low-dose radiotherapy (4 Gy) was proven to be effective for CL with a favourable response and minimal adverse effects [24]. External-beam radiotherapy is a safe and effective therapeutic modality for CL, yielding long-term tumour regression in 96% of cases and stable or improved vision in 76% of cases after a mean follow-up period of 6 years [21]. Moreover, patients with metastatic intraocular lymphoma often require systemic chemotherapy [25]. The prompt improvement in SRD and choroidal thickening after chemotherapy confirmed choroidal involvement in diffuse large B-cell lymphoma, the chief cause of metastatic CL [26].

### 3.2. Combined Diagnosis

Establishing the diagnosis of CL can be challenging, and a definitive diagnosis of malignant lymphoma requires histopathological confirmation. Moreover, the amount of tissue specimen obtained after biopsy of CL is often limited [17], which is attributed to three principal reasons. First, the number of tumour cells in the specimens is small, and they are often mixed with several other non-tumour cells and cell fragments. Second, tumour cells are fragile and easily damaged during surgical operation. Finally, patients often refuse to undergo invasive procedures because of deterioration of their general condition. Therefore, identifying other ocular-specific clinical manifestations and molecular indicators of CL is of immense clinical significance. This report presents a challenging case of CL that was finally confirmed using a combination of ophthalmic examination, aqueous humour detection, and pathological examination.

Several unique imaging features can be used for the diagnosis of lymphoma. B-scan ultrasonography is the most useful diagnostic tool to monitor choroidal thickening and confirm the response to radiotherapy [16]. Localised neuroepithelial detachment without vitritis (observed in our case), which is distinct from vitreoretinal lymphoma, is the hallmark of vitreous cell and subretinal pigment deposition. Local choroidal effusion and partial diffuse thickening of the ciliary body, which are important manifestations of IOL [27], were observed on UBM. EDI-OCT often reveals a hyperreflective signal at the retinal pigment epithelium–Bruch membrane junction, and the neuroepithelial and pigment epithelial layers show wavy changes and choroidal thickening in both eyes [28]. The same findings were also observed on the OCT scan of our patient. In contrast, Hernández-Pons et al. proposed that high-definition OCT findings were not accurate for measuring choroidal thickness, and that retinal changes are non-specific [19]. However, the manifestations of lymphoma on FA and ICGA are specific. First, we found that the precise correspondence of early high fluorescence on the temporal side of the disc and the local high leakage point of the choroid suggest fenestrations of the pigment epithelium caused by depigmentation and focal destruction of the retinal pigment epithelium layer. Second, there was no fluorescence leakage, indicating that the focus was limited to the choroidal layer and the degree of damage to the pigment epithelium was lesser than that in inflammatory diseases. Finally, hypofluorescent spots of varying sizes and distribution may be related to local lymphoma cell aggregation and choroidal circulation disorder. Moreover, Marco et al. also reported that the combination of ICGA and OCT possessed a unique diagnostic value for CL. ICGA depicts choroidal hypopigmentation in the multifocal sub-millimetre region, while OCT depicts diffuse choroidal infiltration [14].

Since CL may be the first manifestation of systemic lymphoma, all patients with suspected lymphoma should undergo systemic evaluation [5,11]. In this case, we immediately examined the routine haematology, tumour markers, and chest, abdominal, and brain CT upon suspicion of ocular lymphoma. We found multiple lymphadenopathies on abdominal CT, which provided the diagnostic basis for CL. In addition, ocular progression should be closely monitored in patients whose systemic symptoms have been controlled, because it may be related to the prognosis [29].

Molecular evaluation of aqueous humour and vitreous specimens is an auxiliary diagnostic modality for CL [30]. Previous studies have reported that the positivity rates of intravitreal IL-10/IL-6 greater than 1.0 were 75% and 90.7% [31,32]. Kimura et al. reviewed 217 patients with IOL and proposed that the IL-10/IL-6 ratio was the most reliable and sensitive index [25]. Although intravitreal IL-10/IL-6 exceeding 1.0 is one of the diagnostic criteria for lymphoma [31], it is noteworthy that the intravitreal IL-10/IL-6 ratio was 0 in our case. However, this result cannot eliminate the existence of lymphoma because the tumour was confined to the choroid; the depth of invasion did not extend beyond the retinal pigment epithelium layer. The distinction from vitreoretinal lymphoma and IL-10/IL-6 ratio >1 are more valuable for the early diagnosis of vitreoretinal lymphoma.

The most confounding entity in the different diagnosis of CL is VKH disease, owing to several similarities between the fundus manifestations of VKH disease and CL that are difficult to distinguish. Two cases of CL masquerading as VKH disease have been reported [24,33]. The presence of neoplastic cells and absence of inflammatory cells in the choroid suggest similarities and differences between haematologic malignancies and inflammatory diseases. In this case, CL was distinguished from VKH disease mainly on the basis of the typical OCT manifestations and combined application of FA and ICGA. First, the OCT manifestations of both conditions are different. VKH lesions involving the retinal and choroidal vessels are characterised by vascular permeability changes, inflammatory infiltration causing choroidal oedema and thickening, and compression of blood vessels, which lead to choroidal circulation disorder. Inflammatory swelling of the choroid with massive infiltration of leukocytes and inflammatory proteins in the exudate on EDI-OCT is a hallmark of acute VKH disease [19]. However, in CL, effusion under the neuroepithelium is homogeneous, without inflammatory proteins and cells, indicating that lymphocyte aggregation blocks microcirculation and transudate formation [24]. In our case, the extent of neuroepithelial detachment was generally bullous and limited and we found that the Bruch membrane was mild, similar to the findings of previous studies. In addition, the combination of FA and ICGA was an effective diagnostic approach. Spot fluorescence leakage around the optic disc is observed in the early stage of VKH disease owing to inflammatory injury, which increases dye accumulation in the late stage [33]. However, a stark difference was observed in our case, i.e., high spot fluorescence around the optic disc at the early stage, without any obvious fluorescence leakage, indicating milder damage to the pigment epithelium than that in VKH disease. The typical ICGA manifestations included low fluorescence dots of uniform size in the middle stage of VKH disease [26], which are different from those of our case. In our patient, ICGA showed persistent low fluorescence spots or flakes of different sizes in the middle and late stages, consistent with the findings of Fukutsu et al. [2]. Our findings provide further evidence for the specific value of ICGA in identifying VKH disease. In conclusion, the characteristic ophthalmic findings on FA and ICGA prompted us to perform further evaluations.

We also considered other diseases that present with choroidal thickening in the differential diagnosis. PR is a retinal degenerative disease caused by primary ocular tumours or metastasis. Bilateral diffuse uveal melanocytic proliferation (BDUMP), a paraneoplastic ocular syndrome occurring in patients with systemic carcinoma, was also considered. The manifestation of BDUMP is similar to that of CL and the two are difficult to distinguish. The fundus is mostly characterised by the presence of multiple round or oval inconspicuous red plaque lesions in the posterior pole. Strong multifocal fluorescence is observed in the early stage on FA [34]. The most significant difference from our case was the presence of pale grey-brown nummular lesions in the fundus and numerous homogeneously sized hypofluorescent areas on FA. However, the size and fewer hypofluorescent lesions in our case differed from to the pale grey lesions seen on FA in BDUMP [35].

We gained some experience and uncovered some merits and demerits of the diagnostic and treatment processes from this case. An incorrect diagnosis can be obtained by solely relying on the results of ophthalmic examination because of the camouflage of symptoms in lymphoma; thus, a combined approach with multiple ophthalmic examinations should be employed for the final diagnosis. Upon reviewing the treatment process of this patient with the haematologist, we realised that administering hormone therapy to enhance immunity, followed by rituximab, may reduce the likelihood of paraneoplastic syndrome. Our study focused on early diagnosis, especially for patients who are initially diagnosed in the ophthalmology department. The possibility of CL should be considered early in the diagnostic process based on the specific manifestations on OCT and other eye examinations. A systemic examination should be performed as soon as possible based on the findings of multiple ophthalmic examinations, which can facilitate early diagnosis and systematic treatment to ensure the patient’s survival.

## 4. Conclusions

Accurate diagnosis and effective timely treatment are essential for CL, owing to the risk of a high degree of malignancy and rapid progression. Furthermore, patients with CL should undergo evaluation to establish an early diagnosis and distinction from systemic lymphoma. Despite the important role of pathological biopsy, a combination of ophthalmologic and systemic examinations and molecular evaluation is crucial. Further studies with larger samples are required to fully evaluate the effectiveness of this combination in the future.

## Figures and Tables

**Table 1:** Detection of Aqueous Humour.

Test Items	Result	Unit	Reference Range	Test Method
IL-2	0	Pg/mL		Cytometric Bead Array
IL-4	0	Pg/mL		Cytometric Bead Array
IL-6	23.1	Pg/mL	1.0~50.0	Cytometric Bead Array
IL-10	0	Pg/mL	0~5.0	Cytometric Bead Array
TNF-α	0	Pg/mL	0~5.0	Cytometric Bead Array
IFN-γ	0.8	Pg/mL	-	Cytometric Bead Array
IL-10/IL-6	0	-	<1	Cytometric Bead Array

## Data Availability

The datasets analysed for this study can be found in the [NAME OF REPOSITORY] https://figshare.com/s/61670599be65e5ca5752, accessed on 1 May 2022.
